# Decreasing proportion of *Anopheles darlingi* biting outdoors between long-lasting insecticidal net distributions in peri-Iquitos, Amazonian Peru

**DOI:** 10.1186/s12936-018-2234-4

**Published:** 2018-02-20

**Authors:** Catharine Prussing, Marta Moreno, Marlon P. Saavedra, Sara A. Bickersmith, Dionicia Gamboa, Freddy Alava, Carl D. Schlichting, Kevin J. Emerson, Joseph M. Vinetz, Jan E. Conn

**Affiliations:** 1 Department of Biomedical Sciences, School of Public Health, University at Albany - State University of New York, Albany, NY USA; 2 Division of Infectious Diseases, Department of Medicine, University of California San Diego, La Jolla, CA USA; 3 Laboratorio ICEMR-Amazonia, Laboratorios de Investigacion y Desarrollo, Facultad de Ciencias y Filosofia, Universidad Peruana Cayetano Heredia, Lima, Peru; 4 Wadsworth Center, New York State Department of Health, Albany, NY USA; 5 Instituto de Medicina Tropical “Alexander von Humboldt”, Universidad Peruana Cayetano Heredia, Lima, Peru; 6 Ministry of Health, Iquitos, Peru; 7 Department of Ecology and Evolutionary Biology, University of Connecticut, Storrs, CT USA; 8 Department of Biology, St. Mary’s College of Maryland, St. Mary’s City, MD USA

**Keywords:** *Anopheles darlingi*, Biting behaviour, Peruvian Amazon, LLINs, Population genetic structure, NextRAD genotyping

## Abstract

**Background:**

In Loreto Department, Peru, a successful 2005–2010 malaria control programme (known as PAMAFRO) included massive distribution of long-lasting insecticidal nets (LLINs). Additional local distribution of LLINs occurred in individual villages, but not between 2012 and 2015. A 2011–2012 study of the primary regional malaria vector *Anopheles darlingi* detected a trend of increased exophagy compared with pre-PAMAFRO behaviour. For the present study, *An. darlingi* were collected in three villages in Loreto in 2013–2015 to test two hypotheses: (1) that between LLIN distributions, *An. darlingi* reverted to pre-intervention biting behaviour; and, (2) that there are separate sub-populations of *An. darlingi* in Loreto with distinct biting behaviour.

**Results:**

In 2013–2015 *An. darlingi* were collected by human landing catch during the rainy and dry seasons in the villages of Lupuna and Cahuide. The abundance of *An. darlingi* varied substantially across years, villages and time periods, and there was a twofold decrease in the ratio of exophagic:endophagic *An. darlingi* over the study period. Unexpectedly, there was evidence of a rainy season population decline in *An. darlingi*. *Plasmodium*-infected *An. darlingi* were detected indoors and outdoors throughout the night, and the monthly *An. darlingi* human biting rate was correlated with the number of malaria cases. Using nextRAD genotyping-by-sequencing, 162 exophagic and endophagic *An. darlingi* collected at different times during the night were genotyped at 1021 loci. Based on model-based and non-model-based analyses, all genotyped *An. darlingi* belonged to a homogeneous population, with no evidence for genetic differentiation by biting location or time.

**Conclusions:**

This study identified a decreasing proportion of exophagic *An. darlingi* in two villages in the years between LLIN distributions. As there was no evidence for genetic differentiation between endophagic and exophagic *An. darlingi*, this shift in biting behaviour may be the result of behavioural plasticity in *An. darlingi*, which shifted towards increased exophagy due to repellence by insecticides used to impregnate LLINs and subsequently reverted to increased endophagy as the nets aged. This study highlights the need to target vector control interventions to the biting behaviour of local vectors, which, like malaria risk, shows high temporal and spatial heterogeneity.

**Electronic supplementary material:**

The online version of this article (10.1186/s12936-018-2234-4) contains supplementary material, which is available to authorized users.

## Background

The main driver of early behavioural resistance in many malaria vectors globally was the extensive reliance on DDT-based indoor residual spraying (IRS). Widespread use of DDT led to modification of behaviour of several vector species that had previously taken blood meals indoors and rested indoors during egg development (endophagy and endophily, respectively), to mostly indoor feeding/outdoor resting (endophagy, exophily), to avoid insecticide exposure [1, 2]. Over the past ~ 10 years, the most effective vector intervention has been long-lasting insecticidal nets (LLINs), which, alone or in combination with IRS, and together with rapid diagnosis and treatment and combination drug therapy, have reduced malaria such that elimination is being considered feasible [3–5]. Despite these advances, primary reliance on LLINs and IRS for vector control has driven physiological resistance to insecticides [6, 7], and behavioural resistance or resilience (e.g., increased exophagy and early evening or daytime biting). Such behavioural modifications have enhanced residual transmission, or transmission that persists despite the reduction of vector populations through control activities [8], including outdoor and non-night-time transmission, in several malaria-endemic areas [9–16], although there are counter-examples [17–19].

It is unclear whether shifting biting behaviour after exposure to LLINs and/or IRS is the result of genetically differentiated populations of anophelines with different feeding behaviours, or of behavioural plasticity, the ability of individuals of the same genotype to adopt different behaviour in response to different environments. Insecticides commonly used for LLINs and IRS have been reported to exert spatial repellent as well as insecticidal effects on anophelines [20]. If anophelines are deterred by the presence of insecticide from feeding successfully at their preferred time or location, they may continue to quest for a blood meal, shifting the overall biting behaviour of the population [13]. If shifts in biting behaviour are instead caused by replacement by genetically different anophelines, these interventions may no longer be as effective against vector populations that have become behaviourally resistant [12]. Evidence for genetic differentiation of anophelines by biting and resting behaviour has been inconclusive: studies have found chromosomal inversion frequency differences between exophagic and endophagic *Anopheles gambiae s.l.* [21, 22] and exophilic and endophilic *Anopheles funestus* [23]; yet studies comparing single nucleotide polymorphisms (SNPs) in putative circadian clock genes between exophagic, endophagic, early and late feeding *Anopheles arabiensis* [24], and whole genome SNPs between exophilic and endophilic *An. arabiensis* [25], detected no genetic differentiation. Understanding whether distinct sub-populations of malaria vectors with different biting behaviour exist could help predict responses to vector control measures and advise vector control programmes as to the most efficient use of their resources.

Except for Venezuela, which in 2015 accounted for an estimated 30% of malaria cases in the Americas [26], Latin American countries have reduced the malaria case load substantially during the last 10 years [26], mainly through rapid case detection and treatment. Generally in this region there is lower coverage of LLINs and IRS compared with endemic areas in Africa and Asia [27–29]. In Peru, the northeastern Loreto Department reports most of the total malaria cases, with an estimated 80% of malaria cases caused by *Plasmodium vivax* [26, 30]. Transmission is seasonal (mainly rainy season, January to June), linked to river levels and mosquito abundance [31–34]. Between 2005 and 2010 in Loreto, the Global Fund’s PAMAFRO (Spanish acronym) initiative strengthened malaria diagnosis and case management, and distributed LLINs, achieving high local coverage, estimated at 98.7% 1 year after distribution in a sub-set of targeted communities [35] and a remarkable monthly malaria case incidence rate below 1/1000 in Peru in 2010–2011, compared to 6–7/1000 in 2005–2006 [36]. However, between 2010 and 2015 (post-PAMAFRO period), there were no widespread LLIN distributions in Loreto, and overall case numbers and the proportion of *Plasmodium falciparum* cases increased [26, 29, 36]. Furthermore, due to the reduced malaria incidence rate, the Peruvian Ministry of Health (MOH) shifted its focus on vector control intervention towards new arboviral outbreaks following the PAMAFRO initiative [36].

The main malaria vector in Latin America, *Anopheles darlingi,* dominates several regions of the Amazon Basin, accounting for > 85% of the anopheline fauna feeding on humans, and much of the malaria transmission, particularly in frontier zones [33, 37–39]. It has successfully invaded human-modified habitats, such as fish ponds, agricultural settlements, highways, mining sites and urban areas [40–44]. This species is behaviourally very plastic, displaying mainly exophily with some reports of endophily (reviewed in [45]), and both endophagy and exophagy (reviewed in [46, 47]), depending on region, season and local environmental variables such as bed-net coverage, house type, and host number and availability [48, 49]. In Amazonian Peru, there are regional records of both endophagic and exophagic behaviour in this species [31, 33, 50]. A previous investigation in peri-Iquitos from 2011 to 2012 found many more exophagic than endophagic *An. darlingi*, although rigorous longitudinal assessment of biting behaviour was not the main focus of that study [34]. To investigate whether there was a modification in *An*. *darlingi*’s feeding behaviour following the end of the PAMAFRO initiative, the present study was designed to quantify the abundance of exophagic *versus* endophagic *An. darlingi* from 2013 to 2015, especially during the 6-month transmission season (~ January–June). Additionally, to test the hypothesis that there are distinct sub-populations of *An. darlingi* with different biting behaviour in peri-Iquitos, a sub-set of collected *An. darlingi* were genotyped using nextRAD (nextera-tagmented, reductively amplified DNA) genotyping-by-sequencing to compare individual exophagic and endophagic mosquitoes biting at different times.

## Methods

### Study area and collection methods

**Fig. 1:**
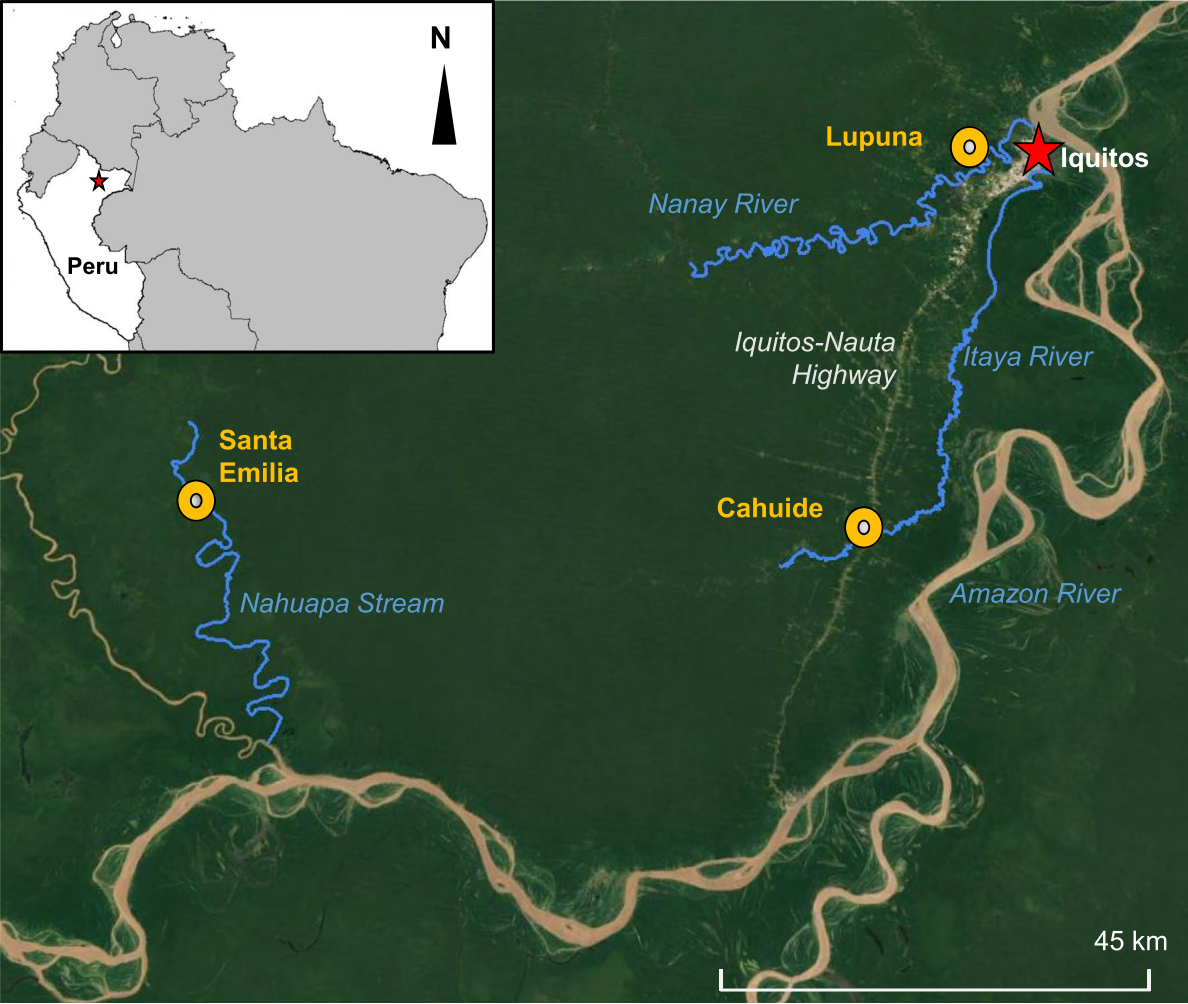
Field study localities in Loreto Department, Peru. Villages of Lupuna (LUP) and Cahuide (CAH) in the peri-Iquitos region, and Santa Emilia (SEM), which is more remote. Iquitos is marked by a star

Adult female *An. darlingi* were collected from three villages in Loreto: San Jose de Lupuna (LUP) (03°44′35.45″S, 73°19′36.91″W) and Cahuide (CAH) (04°13′49.26″S, 73°29′16.20″W) in the peri-Iquitos area, and Santa Emilia (SEM) (04°11′58.99″S, 74°12′20.12″W), a remote site ~ 150 km by river from Iquitos (Fig. 1). Details of these villages are in Moreno et al. [34] and Lainhart et al. [52]. All three sites were part of the PAMAFRO project, which funded comprehensive control activities, particularly LLIN distribution, from 2005 to 2010 [36]. LLINs were distributed twice in LUP and CAH during the PAMAFRO initiative, with the last distributions in October 2010 (CAH) and November 2010 (LUP). In SEM, LLINs were distributed once, in February 2010. Additionally, LLINs were distributed in CAH twice by the International Federation of Red Cross and Red Crescent Societies (IFRC), once in 2012 and once in 2015. Exact dates and coverage for IFRC LLIN distributions are not available. A survey of bed-net coverage in 2012 found that 45% of households in CAH and 88% of households in LUP owned an LLIN [34]. IRS (5% deltamethrin) was conducted sporadically in all three villages between 2012 and 2014 (LUP: August 2012, April 2013, October 2013, December 2014; CAH: May 2012, June 2012, March 2013, November 2014; SEM: March 2012, March 2013, October 2014).

In LUP and CAH, paired collections were conducted indoors and outdoors monthly from January to June (rainy/malaria transmission season) in 2013-2015, and in August, October, and December (dry season) in 2013–2014. In SEM, collections were conducted in January, February and April 2014, and monthly from May–September in 2015 (Additional file 1: Fig. S1). Access to SEM on the Rio Nahuapa (Fig. 1) is difficult, requiring 2 days of travel by boat, and some trips were cancelled due to logistics or flooding issues: in January and February 2014 only outdoor collections were possible, in April 2014 only indoor; but paired outdoor and indoor collections were done monthly from May to September in 2015.

For all collections in LUP and CAH, and 2015 collections in SEM, specimens were collected from a different house each night for two nights/month by human landing catch (HLC) for 12 h (18.00–06.00), using an identical protocol outdoors (peridomestic, within ~ 10 m of each house) and indoors, as previously described in [34]. There were a total of 12 collection hours each indoors and outdoors per night. Individual collectors worked 3 h, then rested 3 h, rotating indoors and outdoors. In SEM in 2014, two collectors worked outside (January and February) or inside (April) at a time for the 12-h collections, for a total of 24 collection hours per night.

Human landing was assumed to result from seeking of blood meals; therefore, the human landing rate was considered equivalent to the human biting rate (HBR), calculated with data obtained from the 12-h collections. Mosquitoes were stored by date, location (village, exophagic/endophagic) and hour of collection. Specimens (nearly exclusively *An. darlingi*) were identified morphologically using entomological keys [53–55]. Mosquitoes were labelled and stored individually with silica gel at room temperature until subsequent analysis.

### Laboratory procedures

#### DNA extraction and *Plasmodium* testing

Genomic DNA from *An. darlingi* specimens collected from LUP, CAH and SEM in 2014–2015 was extracted using the Qiagen DNeasy Blood & Tissue kit (Qiagen, Hilden, Germany), and DNA concentrations were measured using a Qubit Fluorometer (ThermoFisher Scientific, Waltham, MA, USA). Specimens from 2013 were not tested for *Plasmodium* infection due to budgetary constraints. *Plasmodium* infection was detected using real-time PCR of the small sub-unit of the 18S rRNA, with monoplex and triplex TaqMan assays (Life Technologies, Carlsbad, CA, USA), as described in [56]. Pools of heads/thoraces of up to five mosquitoes were analysed for detection of *P. vivax* and *P. falciparum*, and each individual from a positive pool was tested to calculate infection rate (IR = # *An. darlingi* infected with *Plasmodium*/# *An. darlingi* tested) and entomological inoculation rate (EIR = HBR * IR).

#### nextRAD DNA sample preparation

*Anophles darlingi* specimens for nextRAD analysis were selected from among specimens collected from LUP and CAH in March–May 2014 and 2015. For sample selection, the 12-h collection period was split into 4 3-h periods (18.00–21.00, 21.00–00.00, 00.00–03.00, 03.00–06.00). Ten individuals with DNA concentration ≥ 0.5 ng/µL as measured by Qubit were selected from each of the 32 village/year/biting location (exophagic *vs* endophagic)/time period combinations, for a total of 320 mosquitoes. The genomic DNA was sent to SNPsaurus (Institute of Molecular Biology, Eugene, OR, USA), where the samples were genotyped using standard nextRAD genotyping-by-sequencing methods, as in [57]. Briefly, the genomic DNA was fragmented using a Nextera reaction to ligate adapter sequences to the fragments. The fragments were amplified with Nextera primers, and the library was pooled and purified, then size selected to 350–500 bp. The resulting library was then sequenced on an Illumina HiSeq 4000, generating 150 bp reads.

### Statistical analysis

#### Negative binomial regression of *Anopheles darlingi* counts

Analyses of *An. darlingi* counts were conducted on data from the 2013–2015 rainy season (January–June) collections in LUP and CAH (Additional file 2). To account for overdispersion, the count data were analysed by negative binomial regression in R 3.4.1 [58] using the MASS package [59] glm.nb() function. The following independent variables were included: year, site, biting location (exophagic/endophagic), time period (18.00–21.00, 21.00–00.00, 00.00–03.00, 03.00–06.00), and their two-, three- and four-way interactions. Forward selection was used to select variables and interactions for inclusion in the model. The irregular collection schedule for SEM precluded statistical analysis of these data, and LUP and CAH dry season collections were excluded from the analysis because of low collection numbers.

#### Correlation of monthly HBR and human malaria cases

For CAH and LUP in the rainy season in 2013–2015, HBR was aggregated monthly to compare with monthly malaria cases (*P. falciparum* and *P. vivax* combined) reported by the Peruvian MOH. As neither the HBR nor the malaria case distributions were normally distributed, non-parametric Spearman rank correlation was used to assess the relationship between them.

#### nextRAD data analysis

Raw sequence reads were analysed using STACKS v1.75 [60, 61]. Low-quality reads were dropped using the STACKS *process_radtags* program, and retained reads were aligned to the *An. darlingi* genome scaffolds [62] using gsnap [63]. The STACKS *ref_map* pipeline was used to assign genotypes, with the minimum number of reads required to create a stack set at 5, and the STACKS correction module *rxstacks* was used to correct genotype assignments. To increase the quantity of loci and confidence in genotype calls, nextRAD sequencing reads from 57 Brazilian *An. darlingi* previously described in [57] were included to build the catalogue, but excluded in creation of the final SNP database and for subsequent population genetic analysis. The STACKS *populations* program was used to select a single SNP from each locus found in at least 75% of individuals in the dataset. A bash script showing all STACKS parameters used is included as Additional file 3, and the final STRUCTURE dataset used for subsequent analysis is included as Additional file 4.

STRUCTURE analysis [64] was run using the Python program StrAuto, which allows for parallel computation [65]. The STRUCTURE admixture model was run assuming correlated allele frequencies for 20 replicates each of *K* = 1 to 10, with a burn in of 100,000 generations and an MCMC chain of 1,000,000 generations. The Evanno method [66] as implemented in STRUCTURE Harvester [67] was used to determine the optimal value of *K*. CLUMPP v.1.1.2, [68] using the greedy algorithm with random input orders, was used to average the files for each value of *K* shown at the individual level, and distruct v.1.1 [69] was used to create STRUCTURE plots.

Principal components analysis (PCA) was performed using the R ade4 package [70] dudi.pca() function, and PCA plots were created using the R factoextra package [71] fviz_pca_ind() function. Discriminant analysis of principal components (DAPC) [72] was performed using the R package adegenet [73]. In addition, ARLEQUIN v. 3.5.2.2 [74] was used to compute pairwise *F*_*ST*_ values.

## Results

### Heterogeneity of *Anopheles darlingi* biting behaviour

**Fig. 2:**
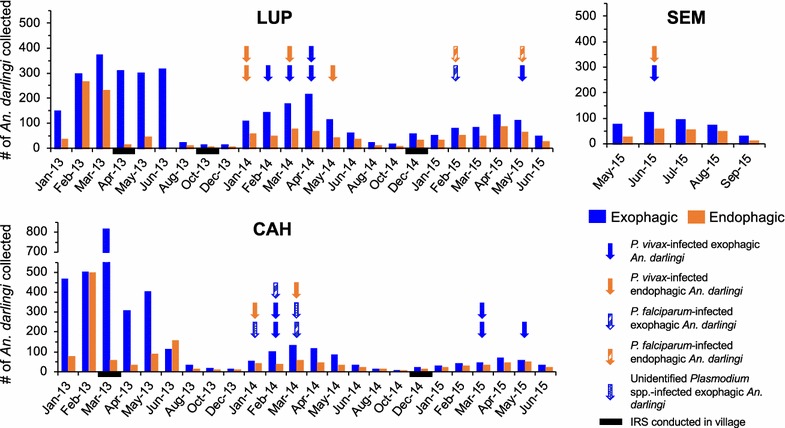
Summary of *Anopheles darlingi* collected monthly, biting outside (exophagic) and inside (endophagic) from 2013 to 2015 in the Peruvian villages of Lupuna (LUP) and Cahuide (CAH), and in 2015 in Santa Emilia (SEM). The month of collection of each *Plasmodium*-infected *An. darlingi* is represented by an arrow, with the colour of the arrow indicating whether the mosquito was exophagic or endophagic and the texture indicating the species of *Plasmodium*. Specimens were not tested for *Plasmodium* in 2013. The months during which IRS was conducted in each village are indicated by black bars

**Fig. 3:**
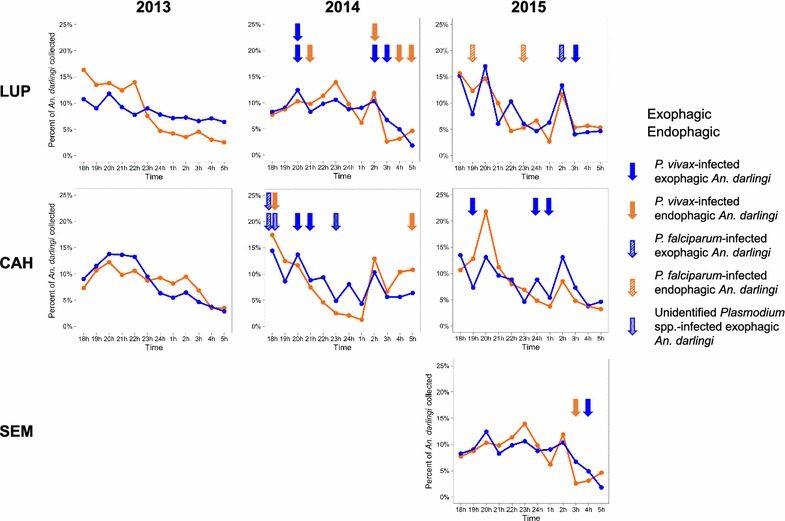
Average proportion of *Anopheles darlingi* collected per hour, biting outside (exophagic) and inside (endophagic), in Lupuna (LUP) and Cahuide (CAH) in 2013–2015 and Santa Emilia (SEM) in 2015. Confidence intervals not shown (for clarity). The hour of collection of each *Plasmodium*-infected *An. darlingi* is represented by an arrow, with the colour of the arrow indicating whether the mosquito was exophagic or endophagic and the texture indicating the species of *Plasmodium*. Specimens were not tested for *Plasmodium* in 2013

The overall number of *An. darlingi* collected from the three localities was 4423 from LUP and 4796 from CAH in 2013–2015 (Additional file 1: Table S1A), 581 from SEM in 2015 (Additional file 1: Table S1B), and 836 from SEM in 2014 (Additional file 1: Table S2). As expected, over 90% of exophagic and endophagic *An. darlingi* were collected in the rainy season, between January and June (Additional file 1: Tables S1, S2, Fig. 2). In all villages and years, more exophagic and endophagic *An. darlingi* were collected before midnight than after (Fig. 3). Most years and villages showed a second peak around 02.00; this is absent in LUP in 2013 in exophagic *An. darlingi*, and very minor in the LUP 2013 endophagic population (Fig. 3).

Of 4561 *An. darlingi* tested for *Plasmodium,* 30 (0.7%) were infected (Table 1). In the three villages, only mosquitoes collected during the rainy season were infected (Fig. 2). More *An. darlingi* were infected with *P. vivax* (n = 23), which is more prevalent in Peru [75, 76], than with *P. falciparum* (n = 5). The *Plasmodium* in two infected *An. darlingi* could not be identified to species (Additional file 1: Table S3). Infected *An. darlingi* were detected biting across the whole 12-h collecting period (Fig. 3). For each locality, the monthly endophagic and exophagic HBR, and the monthly endophagic and exophagic IR and EIR for 2014–2015 collections, are shown in Additional file 1: Tables S1, S2.

**Table 1:** Exophagic and endophagic *Anopheles darlingi Plasmodium* infection rate in Lupuna (LUP), Cahuide (CAH), and Santa Emilia (SEM), 2014–2015

Locality	Year	Exophagic			Endophagic		
		N	No inf	IR	N	No inf	IR
LUP	2014	878	4	0.46	356	4	1.12
	2015	481	2	0.42	293	2	0.68
CAH	2014	510	6	1.18	231	2	0.87
	2015	253	3	1.19	183	0	0.00
SEM	2014	343	3	0.87	493	2	0.41
	2015	361	1	0.28	179	1	0.56

*N* Number tested for *Plasmodium* infection, *No inf* Number of *An. darlingi* infected with *Plasmodium* parasites, *IR* infection rate

**Fig. 4:**
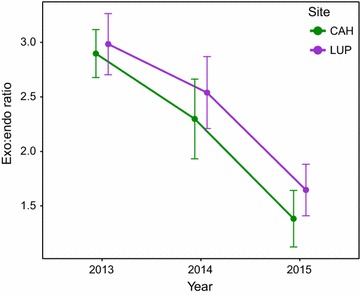
Ratio of *Anopheles darlingi* biting outside (exophagic) to inside (endophagic) per year, aggregated over the rainy season (January–June) in Cahuide (CAH) and Lupuna (LUP), 2013–2015

Negative binomial regression of 8894 *An. darlingi* collected in CAH and LUP during the rainy season in 2013–2015 shows significant differences in counts across years, villages and time periods, and between endophagic and exophagic *An. darlingi* (Table 2). Significant interactions (year X exophagic/endophagic, year X village and year X time period) indicate that these relationships showed considerable context dependence: e.g., CAH counts were much higher than LUP in 2013, but were lower in 2014 and 2015. In both villages, *An. darlingi* counts were higher in 2013 than in 2014–2015 (Fig. 2). More exophagic than endophagic *An. darlingi* were collected in all years in both villages, but the ratio of exophagic to endophagic *An. darlingi* decreased over the study period in both villages, from 2.9 (CAH) and 3.0 (LUP) in 2013 to 1.4 (CAH) and 1.6 (LUP) in 2015 (Fig. 4). As a comparison, in SEM, the exophagic:endophagic ratio in 2015 was 2.0 (Additional file 1: Table S1B), although the months of collection were different from those in CAH and LUP. Significant differences in *An. darlingi* counts were also found among 3-h collection time periods, with overall higher biting from 18.00–21.00 to 21.00–00.00 in 2013, and from 18.00–21.00 in 2014–2015, than during the other time periods. Non-parametric Kruskal–Wallis analysis of the count dataset produced comparable results to the negative binomial regression (Additional file 1: Table S4).

**Table 2:** Negative binomial regression of abundance of *Anopheles darlingi*, Cahuide and Lupuna, 2013–2015 rainy season (January–June)

Variable	*β*	*e* ^*β*^	SE	*p* value
Intercept	3.24	25.42	0.14	< 0.0001
Exophagic/endophagic (reference = endophagic)				
Exophagic	1.11	3.05	0.11	< 0.0001
Year (reference = 2013)				
2014	− 1.30	0.27	0.20	< 0.0001
2015	− 1.45	0.24	0.20	< 0.0001
Time period (reference = 18.00–21.00)				
21.00–00.00	− 0.11	0.90	0.16	0.4784
00.00–03.00	− 0.56	0.57	0.16	0.0004
03.00–06.00	− 0.93	0.39	0.16	< 0.0001
Village (reference = Cahuide)				
Lupuna	− 0.41	0.66	0.11	0.0002
Year × exophagic/endophagic				
2014 Exophagic	− 0.20	0.82	0.16	0.2241
2015 Exophagic	− 0.67	0.51	0.17	< 0.0001
Year × village				
2014 Lupuna	0.84	2.31	0.16	< 0.0001
2015 Lupuna	0.97	2.62	0.17	< 0.0001
Year × time period				
2014 21.00–00.00	− 0.58	0.56	0.23	0.0115
2015 21.00–00.00	− 0.46	0.63	0.23	0.0451
2014 00.00–03.00	− 0.13	0.88	0.23	0.5769
2015 00.00–03.00	− 0.03	0.97	0.23	0.9116
2014 03.00–06.00	0.25	1.28	0.23	0.2838
2015 03.00–06.00	− 0.11	0.89	0.24	0.6294

*β* Regression coefficient, *e*^*β*^ Exponentiated regression coefficient, *SE* Standard error

### Correlation between monthly HBR and human malaria cases

The number of yearly malaria cases of *P. vivax* and *P. falciparum* from 2010 to 2016 in LUP (population = 432; 2014 human census), CAH (population = 910; 2014 human census), and SEM (population = 212; 2014 human census) is depicted in Additional file 1: Fig. S2, demonstrating substantial fluctuation in cases in all three villages, and a distinct temporal pattern in each village, with increasing *P. falciparum* in LUP. In LUP and CAH, the monthly HBR of exo- and endophagic *An. darlingi* combined was moderately but significantly correlated with the number of malaria cases in the same locality in the current month (Additional file 1: Fig. S3, *ρ* = 0.55, *p* = 0.0005) and the previous month (*ρ* = 0.45, *p* = 0.004). The exophagic HBR was more highly correlated with malaria cases in the current month (*ρ* = 0.548, *p* = 0.0005) than the endophagic HBR (*ρ* = 0.344, *p* = 0.040).

### No evidence of population genetic structure of *Anopheles darlingi* by biting behaviour

From the 320 nextRAD-genotyped *An. darlingi*, an average of 1,912,124 (range: 2712–17,342,438) reads per sample passed quality filtering, and an average of 1,007,380 (range 701–8,124,687) reads per sample were aligned to *An. darlingi* reference genome scaffolds. To increase the number of loci in the final analysis, only samples with at least 200,000 aligned reads (n = 162) were included for genotyping. Genotypes were called at an average of 58,480 (SD 65,548) loci per sample. Within individuals, 12.92% (SD 6.11%) loci were polymorphic (Additional file 5). The final SNP dataset includes one biallelic SNP from each locus genotyped in at least 75% of the 162 individual mosquitoes, a total of 1021 loci (Additional file 4). The average sequencing depth across all 1021 loci and 162 individuals was 96X.

**Fig. 5:**
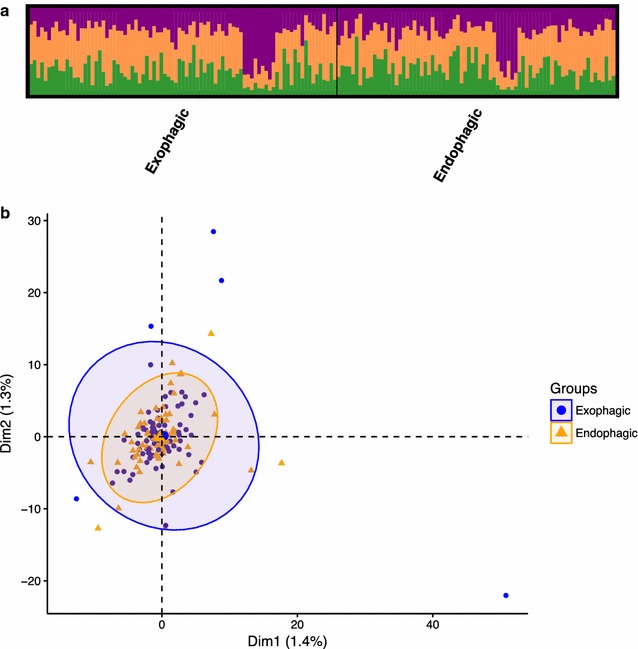
Results of STRUCTURE and PCA of 1021-locus SNP dataset, comparing endophagic and exophagic *Anopheles darlingi*. **a** STRUCTURE results depicting three inferred genetic clusters. Although the proportion of membership in each cluster varies across individual *An. darlingi*, all individuals have non-zero membership in all three clusters, indicating admixture and no significant structuring. **b** PCA, with colours reflecting endophagic *vs* exophagic individuals

STRUCTURE and STRUCTURE Harvester analyses of the SNP dataset supported 3 genetic clusters (optimal *K* = 3); however, there was not a clear peak for the value of *ΔK* for *K* = 2–9 (*ΔK* = 10 for *K* = 3), and the estimated natural log probability of the data (lnPr(X|K)) was very similar for *K* = 1 through 8 (Additional file 1: Fig. S4A, B). The Evanno method is not able to find the optimal *K* if *K* = 1 [66]. In the STRUCTURE plot for *K* = 3, all individuals are admixed among the three clusters, regardless of biting location (Fig. 5a), collection village/year (Additional file 1: Fig. S5D), or biting time (Additional file 1: Fig. S5E), indicating that all 162 individuals belong to a single population. The STRUCTURE plots for *K* = 2 also support this lack of population genetic structure (Additional file 1: Fig. S5A–C).

PCA of the SNP dataset was consistent with a single homogeneous population, with no separation by biting location (Fig. 5b), collection village/year (Additional file 1: Fig. S6A), or biting time (Additional file 1: Fig. S6B). Similarly, the Bayesian information criterion (BIC) of the *k*-means clustering algorithm implemented in preparation for DAPC indicated that the optimal number of clusters was 1 (Additional file 1: Fig. S4C). Pairwise *F*_*ST*_ values indicated low genetic differentiation of exophagic and endophagic *An. darlingi* (*F*_*ST*_ = 0.0016, *p* = 0.62); *An. darlingi* at different biting times (all pairwise *F*_*ST*_ values < 0.005 with *p* > 0.40); and *An. darlingi* from different collection villages/years (CAH 2014 vs CAH 2015 *F*_*ST*_ = 0.0149, *p* < 0.05; all other pairwise *F*_*ST*_ values < 0.001 with *p* > 0.88) (Additional file 1: Table S5A–C).

## Discussion

This study detected, across 3 years, a shift in *An. darlingi* in LUP and CAH towards decreased exophagy. This shift occurred between distributions of LLINs in these villages; the most recently distributed LLINs in CAH were a year old and in LUP were over 2 years old by the start of this study, and LLINs in both villages were at least 3 years old (the expected lifetime of LLINs [51]) by the end of the study. It is therefore possible that this shift represents a return to baseline biting behaviour in these villages following a previous shift towards increased exophagy driven by LLIN exposure.

IRS was conducted in CAH and LUP sporadically during this study. However, *An. darlingi* is known to rest mainly outdoors following blood feeding [47], so it is unlikely that IRS is effective against this vector. In this study, there was not a consistent effect of IRS on the *An. darlingi* abundance or exophagic:endophagic ratio in either the month IRS was conducted or the following month in these two villages (Fig. 2).

This study further confirms the heterogeneous biting behaviour of *An. darlingi* [34, 43, 47, 77–79]. A range of local environmental or ecological changes can influence the ratio of exo/endophagic *An. darlingi.* For example, in a gold mining area in Venezuela, *An. darlingi* was found to be significantly more exo- than endophagic, attributed mainly to the location of villages within forested areas, and to houses with incomplete walls [42]. A similar pattern of high exophagy/low endophagy in *An. darlingi* was detected along a corridor of a highway deforested for power line construction in Porto Velho, Rondonia state, Brazil [80].

Shifts in behaviour in vector anophelines towards increased exophagy following LLIN distribution are relatively common [9–11, 14]. In these studies, such shifts were seen within 1 year after LLIN distribution, or during periods of high LLIN usage. Shifts in biting behaviour can result from physiological (insecticide-induced) or behavioural resistance, which can be difficult to distinguish [12]. Because there is little documented physiological resistance in *An. darlingi*, including populations sampled for this study [81], the documented exophagic/endophagic shift may be evidence for behavioural resistance that emerged as a result of LLIN usage. However, some previously reported shifts may be the result of changes in species composition, or plasticity in feeding responses [2, 13]. For example, using mark-release-recapture, individual *Anopheles farauti* were found to feed both outdoors and indoors [19].

In the current study, using 1021 genome-wide SNPs, there was no evidence of genetic differentiation between exophagic and endophagic *An. darlingi*, or among *An. darlingi* biting at different times during the night. These results were consistent across both model-based (STRUCTURE) and non-model-based analyses (PCA/DAPC). In addition, thorough exploration of the parameter space, by changing the exclusion criteria for low coverage samples and the STACKS settings for filtering of loci, consistently returned evidence that all samples belonged to a single homogeneous population. The lack of detectable population structural differences among the *An. darlingi* from this study suggests that the reported shifts in biting behaviour are due to behavioural plasticity resulting from reduced spatial repellence of aging LLINs, rather than genetically differentiated populations of exophagic and endophagic *An. darlingi*. However, the lack of population genetic structure does not preclude a genetic basis for changes in *An. darlingi* biting behaviour. It is possible that the methods used were unable to detect smaller-scale genetic differences between exophagic and endophagic *An. darlingi* that do not influence the overall genetic structure.

Previous studies have found microgeographic genetic differentiation between *An. darlingi* by habitat [52] and season [77]. In addition, a recent study using whole-genome SNPs found genetic differentiation between *An. darlingi* collected in two rural Brazilian villages ~ 60 km apart, which had experienced different levels of deforestation [82]. Although these Brazilian villages were approximately the same distance apart as the villages in the current study, it is not surprising that there was no evidence of genetic structure between *An. darlingi* collected in LUP and CAH, because both are riverine villages with similar ecological characteristics [34, 52].

Across its broad distribution, *An. darlingi* populations exhibit a wide range of peak biting times and patterns (unimodal, bimodal, trimodal, no peak) [47, 83, 84]. Furthermore, in a study in Amapá state, northern Amazonian Brazil, Voorham [85] found intra-population variation of biting activity in *An. darlingi* to be as high as inter-population variation. Some variation is attributed to seasonality [50, 77, 86], and some is assumed to be the result of interaction between local ecological and endogenous factors [45]. The current study determined that in the peri-Iquitos area, more *An. darlingi* were biting before midnight than afterwards, especially early in the evening, in agreement with observations in previous studies in Peru [32–34] and some regions of Brazil [43, 78]; although another Brazilian study found *An. darlingi* biting throughout the night [84]. A preponderance of early evening biting is likely related to the availability of humans as hosts while they are engaged in various activities prior to retiring under bed nets.

There was a second, smaller biting peak around 02.00 in all years and villages except 2013 in the exophagic individuals from LUP (Fig. 3). Although there were no genetic structural differences by biting time, it is possible that there are individual genes determining biting time in the population that do not influence the overall genetic structure (as investigated in [24]). Alternatively, this additional peak may be the result of phenotypic plasticity in biting behaviour within the population.

This study shows similar overall patterns in abundance, HBR, and biting behaviour of *An. darlingi* for LUP and CAH between 2013 and 2015. That the two communities are ~ 60 km apart and located on different rivers suggests that these populations may respond to some types of regional environmental or anthropogenic change as a single metapopulation. In 2012 [34], peak monthly HBRs of exophagic populations of *An. darlingi* were similar in LUP and CAH, and by 2015, they had declined similarly. In addition, the changing ratio of exophagic:endophagic *An. darlingi* from year-to-year is quite congruent (Fig. 4). However, as the negative binomial regression results demonstrate, local context strongly influences the patterns of abundance in these populations of *An. darlingi*. In summary, there is evidence for both metapopulation and local population behavioural patterns in *An. darlingi* in Loreto, but the mechanisms involved have not yet been identified.

The aggregated monthly HBR of *An. darlingi* (exophagic, endophagic and both together) was significantly correlated with monthly malaria case numbers in LUP and CAH. This is particularly interesting because cases of *P. falciparum* have increased since 2015, especially in LUP, representative of a wider regional trend in Loreto [29, 87], whereas *P. vivax* cases peaked in LUP in 2014, and CAH experienced a major *P. vivax* outbreak in 2012–2013 after which cases have fluctuated considerably (Additional file 1: Fig. S2). These data confirm that the HBR and malaria incidence are highly related, though it is clear that other parameters used in the calculation of vectorial capacity, such as vector survival rates [88], are also valuable for predicting malaria risk.

Although in the present study the overall numbers of infected *An*. *darlingi* (n = 30) were insufficient for statistical analysis, there were endophagic and exophagic *An. darlingi* infected with both *P. vivax* and *P. falciparum* throughout the rainy season (January–June), before and after midnight. Thus, during the rainy season, villagers are at risk of malaria infection both inside their houses and in the peridomestic area throughout the night.

An explanation for the decline in the rainy season *An. darlingi* population sizes in LUP and CAH over time is elusive. A massive flood in Loreto in April 2012 [89], attributed mainly to an early La Niña event [90], may have influenced survival or population dynamics, perhaps by destruction of breeding sites. Less massive flooding in Suriname, combined with several vector interventions and malaria case management, reduced malaria incidence to near zero, in a region where *An. darlingi* was the principal vector [91]. In the village of LUP, there was no discernible immediate effect of the flood on the peak HBR in April–May between 2011 (before the flood) and 2012 (immediately after the flood [34]), although longer term effects cannot be ruled out.

Modelling has demonstrated that *P. falciparum* is likely more sensitive than *P. vivax* to changes in malaria vector survival rates due to longer sporogonic cycle duration [88]. If *An. darlingi* survival rates had been measured over the 3 years of this study, the relationship between survival and the increase in *P. falciparum* cases could have been investigated. In addition, as this study analysed only *An. darlingi* collected by HLC, it is possible that a sub-population of *An. darlingi*, not sampled in this study because it feeds mainly on animals, also contributes to malaria transmission. Although a previous analysis of blood meal sources in resting *An. darlingi* in LUP, CAH and SEM found that the majority of mosquitoes tested had fed on humans (human blood index (HBI): 0.58–0.87), with a similar infection rate to that found in the current study (0.42%) [49], a substantial proportion of *An. darlingi* feed on non-human hosts in this region. It is unknown whether *An. darlingi* feeding on different hosts are genetically distinct, as has been demonstrated in *An. arabiensis* in Tanzania [25]. Another limitation of this study was the sporadic collections at SEM, which prohibited statistical and genetic comparisons with LUP and CAH. Finally, this study did not determine whether LLINs were used or IRS was conducted in the individual houses in which collections were conducted. However, collections rotated between different houses on different nights throughout the study in an effort to obtain an unbiased, representative sampling of *An. darlingi* biting behaviour in the villages during the study period.

## Conclusions

This study identified a decreasing proportion of outdoor biting among *An. darlingi* in the years following LLIN distributions in two villages in Amazonian Peru. The results strongly suggest that LLINs (to be replaced every ~ 3 years [51]) would reduce endophagic malaria transmission risk even where *An. darlingi* is also exophagic. Controlling exophagic malaria transmission, on the other hand, is extremely challenging [92]. Potential solutions include use of genetically modified mosquitoes, personal and/or spatial repellents, insecticide treatment of livestock, insecticidal sugar baits and perhaps, under certain circumstances, treatment of asymptomatic persons. Lastly, given the tremendous temporal and spatial heterogeneity of *An. darlingi*, the context of each village needs to be considered in planning malaria control programmes, highlighting the need for continuous monitoring of vector abundance and behaviour in malaria-endemic areas, particularly after changes in vector control interventions.

## Additional files

**Additional file 1: Table S1A.** Monthly abundance, HBR, IR, EIR of *Anopheles darlingi* from Cahuide and Lupuna, 2013–2015. **Table S1B.** Monthly abundance, HBR, IR, EIR of *Anopheles darlingi* from Santa Emilia, 2015. **Table S2.** Monthly abundance, HBR, IR, EIR of *Anopheles darlingi* from Santa Emilia, 2014. **Table S3.** Infection of *Anopheles darlingi* by year, month, time, exophagic *versus* endophagic, village and *Plasmodium* species. **Table S4.** Kruskal-Wallis analysis on ranked abundance of *Anopheles darlingi*, Cahuide and Lupuna, 2013–2015 rainy season (January-June). **Table S5.** Pairwise *F*_*ST*_ values comparing *Anopheles darlingi* by (A) year/locality, (B) exophagic/endophagic, and (C) collection time. **Figure S1.** Schedule of *Anopheles darlingi* human landing catch collections in Lupuna, Cahuide, and Santa Emilia, 2013–2015. **Figure S2.** Number of reported human cases of *Plasmodium vivax* and *Plasmodium falciparum* in Cahuide, Lupuna, and Santa Emilia, 2010–2016. **Figure S3.** Monthly human biting rate plotted against the monthly number of malaria cases in Cahuide and Lupuna, rainy season, 2013–2015. **Figure S4.** Estimation of number of clusters in SNP dataset. **Figure S5.** Results of STRUCTURE analysis of SNP dataset for *K*=2 and *K*=3, with individual *Anopheles darlingi* ordered by locality/year, exophagic/endophagic, and collection time period. **Figure S6.** Results of PCA of SNP dataset, with individual *Anopheles darlingi* coloured by locality/year and collection time period.

**Additional file 2.** Dataset of *Anopheles darlingi* counts by collection, month, year, site, exophagic/endophagic, and time period. Collections in Cahuide and Lupuna from 2013-2015 and in Santa Emilia from 2015 are included.

**Additional file 3.** Bash scripts with commands used to align the sequencing reads to the *Anopheles darlingi* genome scaffolds and to run the Stacks pipeline.

**Additional file 4.** STRUCTURE file used for STRUCTURE, PCA and DAPC analyses, with genotypes for 162 individuals at 1,021 loci.

**Additional file 5.** nextRAD sample collection information, number of sequence reads, and unique stacks genotyped. Includes collection and sequencing details for all 320 individuals sequenced, and Stacks details for 162 individuals included in Stacks and downstream analyses.

## Abbreviations

BIC: Bayesian information criterion
CAH: Cahuide
DAPC: discriminant analysis of principal components
EIR: entomological inoculation rate
HBI: human blood index
HBR: human biting rate
HLC: human landing catch
IR: infection rate
IRS: indoor residual spray
LLINs: long-lasting insecticidal nets
LUP: Lupuna
MOH: Peruvian Ministry of Health
nextRAD: nextera-tagmented, reductively-amplified DNA
PAMAFRO: Global Fund programme to control malaria in Andean country border areas (Spanish acronym)
PCA: principal components analysis
SEM: Santa Emilia
SNP: single nucleotide polymorphism
