# Supplementary material for: Decreasing proportion of Anopheles darlingi biting outdoors between long-lasting insecticidal net distributions in peri-Iquitos, Amazonian Peru
Source: Malar J. 2018 Feb 20;17:86. doi: 10.1186/s12936-018-2234-4 (PMC5819687; doi:10.1186/s12936-018-2234-4)

Additional File 1: Supplementary Tables and Figures

Decreasing proportion of *Anopheles darlingi* biting outdoors between long-lasting insecticidal net distributions in peri-Iquitos, Amazonian Peru

Catharine Prussing, Marta Moreno, Marlon P. Saavedra, Sara A. Bickersmith, Dionicia Gamboa, Freddy Alava, Carl D. Schlichting, Kevin J. Emerson, Joseph M. Vinetz, Jan E. Conn

Table S1A. Monthly abundance, HBR, IR, and EIR of exophagic and endophagic *Anopheles darlingi*, Cahuide and Lupuna, 2013-2015

	LUP								CAH							
**Yr/Mo**	**Exo**	**HBR (±SE)**	**IR**	**EIR**	**Endo**	**HBR (±SE)**	**IR**	**EIR**	**Exo**	**HBR (±SE)**	**IR**	**EIR**	**Endo**	**HBR (±SE)**	**IR**	**EIR**
**2013**																
Jan	147	73.5 (±0.5)			33	16.5 (±2.5)			466	233 (±121)			74	37 (±32)		
Feb	295	147.5 (±37.5)			264	132 (±64)			501	250.5 (±141.5)			497	248.5 (±165.5)		
Mar	372	186 (±31)			230	115 (±21)			814	407 (±113)			55	27.5 (±7.5)		
Apr	307	153.5 (±36.5)			10	5 (±5)			305	152.5 (±86.5)			32	16 (±8)		
May	300	150 (±37)			44	22 (±3)			402	201 (±21)			86	43 (±14)		
Jun	313	156.5 (±58.5)			0	0 (±0)			111	55.5 (±29.5)			153	76.5 (±76.5)		
Aug	20	10 (±3)			8	4 (±1)			29	14.5 (±3.5)			11	5.5 (±1.5)		
Oct	11	5.5 (±0.5)			3	1.5 (±1.5)			15	7.5 (±0.5)			5	2.5 (±0.5)		
Dec	10	5 (±2)			3	1.5 (±1.5)			12	6 (±2)			5	2.5 (±0.5)		
**Total**	**1775**				**595**				**2655**				**918**			
**2014**																
Jan	105	52.5 (±9.5)	0.00	0.00	57	28.5 (±6.5)	3.64	1.04	50	25 (±5)	2.13	0.53	38	19 (±7)	2.70	0.51
Feb	140	70 (±4)	0.72	0.51	47	23.5 (±3.5)	0.00	0.00	97	48.5 (±4.5)	3.16	1.53	36	18 (±3)	0.00	0.00
Mar	176	88 (±6)	0.57	0.51	75	37.5 (±7.5)	1.37	0.51	131	65.5 (±28.5)	1.59	1.04	53	26.5 (±3.5)	2.00	0.53
Apr	215	107.5 (±6.5)	0.95	1.02	66	33 (±1)	0.00	0.00	113	56.5 (±12.5)	0.00	0.00	42	21 (±3)	0.00	0.00
May	111	55.5 (±15.5)	0.00	0.00	39	19.5 (±4.5)	2.63	0.51	81	40.5 (±7.5)	0.00	0.00	30	15 (±3)	0.00	0.00
Jun	58	29 (±3)	0.00	0.00	33	16.5 (±2.5)	0.00	0.00	29	14.5 (±8.5)	0.00	0.00	19	9.5 (±1.5)	0.00	0.00
Aug	21	10.5 (±2.5)	0.00	0.00	8	4 (±1)	0.00	0.00	10	5 (±2)	0.00	0.00	9	4.5 (±0.5)	0.00	0.00
Oct	15	7.5 (±1.5)	0.00	0.00	6	3 (±1)	0.00	0.00	3	1.5 (±1.5)	0.00	0.00	4	2 (±0)	0.00	0.00
Dec	56	28 (±2)	0.00	0.00	31	15.5 (±1.5)	0.00	0.00	20	10 (±1)	0.00	0.00	10	5 (±1)	0.00	0.00
**Total**	**897**				**362**				**534**				**241**			
**2015**																
Jan	48	24 (±1)	0.00	0.00	32	16 (±5)	0.00	0.00	26	13 (±1)	0.00	0.00	19	9.5 (±1.5)	0.00	0.00
Feb	78	39 (±0)	1.33	0.52	49	24.5 (±0.5)	2.13	0.52	38	19 (±1)	0.00	0.00	27	13.5 (±2.5)	0.00	0.00
Mar	81	40.5 (±14.5)	0.00	0.00	46	23 (±9)	0.00	0.00	44	22 (±1)	4.76	1.05	31	15.5 (±2.5)	0.00	0.00
Apr	132	66 (±4)	0.00	0.00	85	42.5 (±0.5)	0.00	0.00	68	34 (±3)	0.00	0.00	44	22 (±1)	0.00	0.00
May	109	54.5 (±5.5)	0.93	0.51	63	31.5 (±3.5)	1.64	0.52	55	27.5 (±4.5)	1.82	0.50	48	24 (±4)	0.00	0.00
Jun	46	23 (±3)	0.00	0.00	25	12.5 (±1.5)	0.00	0.00	29	14.5 (±5.5)	0.00	0.00	19	9.5 (±3.5)	0.00	0.00
**Total**	**494**				**300**				**260**				**188**			

Exo: exophagic; Endo: endophagic; HBR: Human Biting Rate; HBR represents the average bites per person per night (b/p/n) obtained from a mean of two days/12 hours per day per collection month (1 collector each inside and outside per night); IR: infection rate; EIR: entomological inoculation rate. IR and EIR not available for 2013.

Table S1B. Monthly abundance, HBR, IR, and EIR of exophagic and endophagic *An. darlingi*, Santa Emilia, 2015

Month	Exo	HBR (±SE)	IR	EIR	Endo	HBR (±SE)	IR	EIR
May	75	37.5 (±11.5)	0.00	0.00	26	13 (±6)	0.00	0.00
Jun	121	60.5 (±13.5)	0.84	0.51	57	28.5 (±0.5)	1.75	0.50
Jul	93	46.5 (±1.5)	0.00	0.00	52	26 (±6)	0.00	0.00
Aug	70	35 (±2)	0.00	0.00	48	24 (±3)	0.00	0.00
Sep	28	14 (±1)	0.00	0.00	11	5.5 (±5.5)	0.00	0.00
**Total**	**387**				**194**			

Exo: exophagic; Endo: endophagic; HBR: Human Biting Rate; IR: infection rate; EIR: entomological inoculation rate

Table S2. Monthly abundance, HBR, IR, and EIR of *An. darlingi* from Santa Emilia, 2014

Mo-Yr	Exo/Endo	No. Collected	HBR (±SE)	No. inf.	IR	EIR
Jan-2014	Exo	100	25.00 (±13.0)	0	0.00	0.00
Feb-2014	Exo	243	60.75 (±41.3)	3	1.23	0.75
Apr-2014	Endo	493	123.25 (±95.3)	2	0.41	0.50

Exo: Exophagic, Endo: Endophagic; HBR: Human Biting Rate; No. inf.: number infected with *Plasmodium* parasites; IR: infection rate; EIR: entomological inoculation rate. HBR represents the average bites per person per night (b/p/n) obtained from a mean of two days/12 hours per day per collection month (2 collectors per night).

Table S3. Infection of *An. darlingi* by year, month, time, exophagic vs. endophagic, village and *Plasmodium* species

Year	Locality	Exo/endo	Time	Month	*Plasmodium* spp.	Total N
2014	LUP	Endo	21:00	Jan	*P. vivax*	1234
		Endo	5:00	Jan	*P. vivax*	
		Exo	20:00	Feb	*P. vivax*	
		Endo	2:00	Mar	*P. vivax*	
		Exo	20:00	Mar	*P. vivax*	
		Exo	2:00	Apr	*P. vivax*	
		Exo	3:00	Apr	*P. vivax*	
		Endo	4:00	May	*P. vivax*	
	CAH	Endo	5:00	Jan	*P. vivax*	741
		Exo	18:00	Jan	*Plasmodium spp.*	
		Exo	20:00	Feb	*P. vivax*	
		Exo	21:00	Feb	*P. vivax*	
		Exo	18:00	Feb	*P. falciparum*	
		Exo	23:00	Mar	*Plasmodium spp.*	
		Exo	18:00	Mar	*P. falciparum*	
		Endo	18:00	Mar	*P. vivax*	
	SEM	Exo	21:00	Feb	*P. vivax*	836
		Exo	23:00	Feb	*P. vivax*	
		Exo	24:00	Feb	*P. vivax*	
		Endo	22:00	Apr	*P. vivax*	
		Endo	3:00	Apr	*P. vivax*	
2015	LUP	Exo	2:00	Feb	*P. falciparum*	774
		Endo	19:00	Feb	*P. falciparum*	
		Endo	23:00	May	*P. falciparum*	
		Exo	3:00	May	*P. vivax*	
	CAH	Exo	24:00	Mar	*P. vivax*	436
		Exo	1:00	Mar	*P. vivax*	
		Exo	19:00	May	*P. vivax*	
	SEM	Endo	3:00	Jun	*P. vivax*	540
		Exo	4:00	Jun	*P. vivax*	

CAH: Cahuide; LUP: Lupuna; SEM: Santa Emilia; Exo: exophagic; Endo: endophagic

Table S4. Kruskal-Wallis analysis on ranked abundance of *An. darlingi*, Cahuide and Lupuna, 2013-2015 rainy season (January-June)

	Degrees of freedom	F-value	P-value
Exophagic/Endophagic	1	237.85	<0.0001
Year	2	73.47	<0.0001
Time period	3	36.54	<0.0001
Village	1	24.02	<0.0001
Year X Exophagic/Endophagic	2	23.14	<0.0001
Year X Village	2	11.21	<0.0001
Village X Exophagic/Endophagic	1	6.05	0.014
Exophagic/Endophagic X Time period	3	5.06	0.002

*Time periods: 6-9pm, 9pm-12am, 12-3am, 3-6am

Analysis of *An. darlingi* counts was conducted on data for the rainy season (January-June) collections in LUP and CAH for 2013-15. Because these data could not be transformed to meet ANOVA assumptions of normality or homoscedasticity, count data were ranked, ties were resolved by averaging, and non-parametric Kruskal-Wallis analysis was performed on the ranks. The following independent variables were included: year, site, exo/endo, time period (the 12 hour collections were split into four three-hour periods: 6-9pm, 9pm-12am, 12-3am, and 3-6am), and their two-, three- and four-way interactions. All non-significant interactions were dropped from the final analysis.

Table S5A. Pairwise F_{ST} values comparing *An. darlingi* by year and locality

	**CAH 2014**	**LUP 2014**	**CAH 2015**
**LUP 2014**	-0.0037		
**CAH 2015**	0.0149*	-0.0094	
**LUP 2015**	-0.0197	-0.0628	0.0005

* $p < 0.05$ ($p > 0.88$ for all other comparisons)

Table S5B. Pairwise F_{ST} value comparing exophagic and endophagic *An. darlingi* ($p = 0.62$)

	**Exophagic**
**Endophagic**	0.0016

Table S5C. Pairwise F_{ST} value comparing *An. darlingi* by collection time ($p > 0.40$ for all comparisons)

	**6pm-9pm**	**9pm-12am**	**12am-3am**
**9pm-12am**	0.0043		
**12am-3am**	0.0038	0.0024	
**3am-6am**	0.0046	-0.0048	0.0048

Figure S1. Schedule of *An. darlingi* human landing catch (HLC) collections in Lupuna, Cahuide, and Santa Emilia, 2013-2015

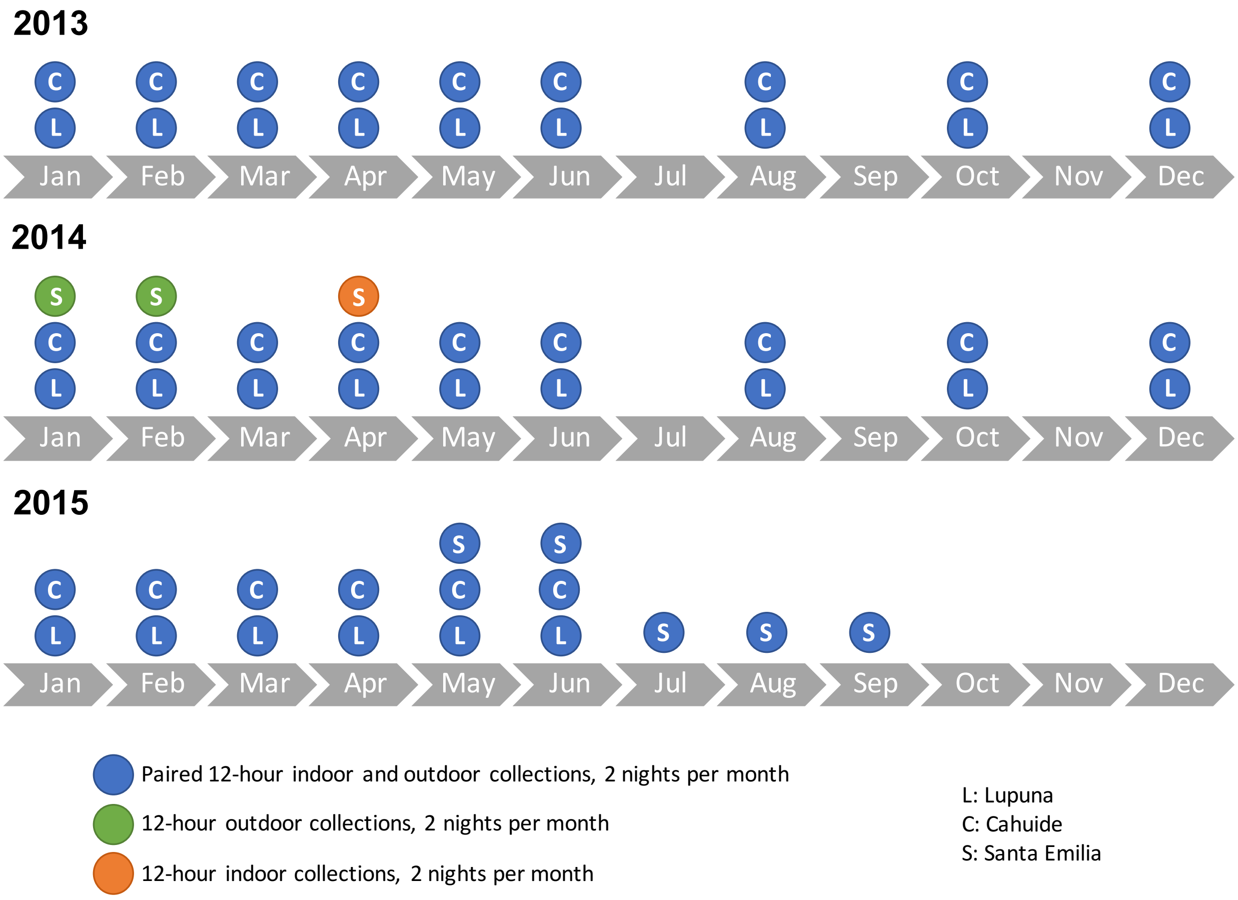

Figure S2. Number of reported human cases of *Plasmodium vivax* (green lines) and *Plasmodium falciparum* (purple lines) in Cahuide, Lupuna, and Santa Emilia, 2010-2016

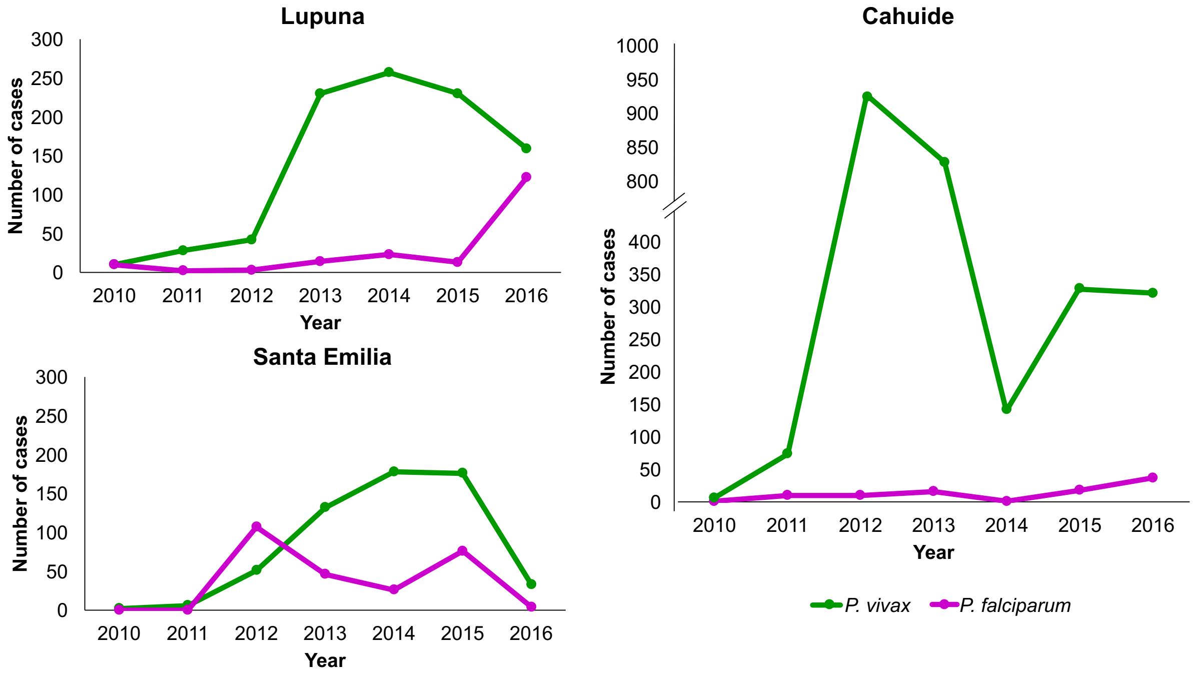

Figure S3. Monthly human biting rate (HBR) plotted against the monthly number of malaria cases (*Plasmodium vivax* and *Plasmodium falciparum* combined) in Cahuide and Lupuna, rainy season, 2013-2015

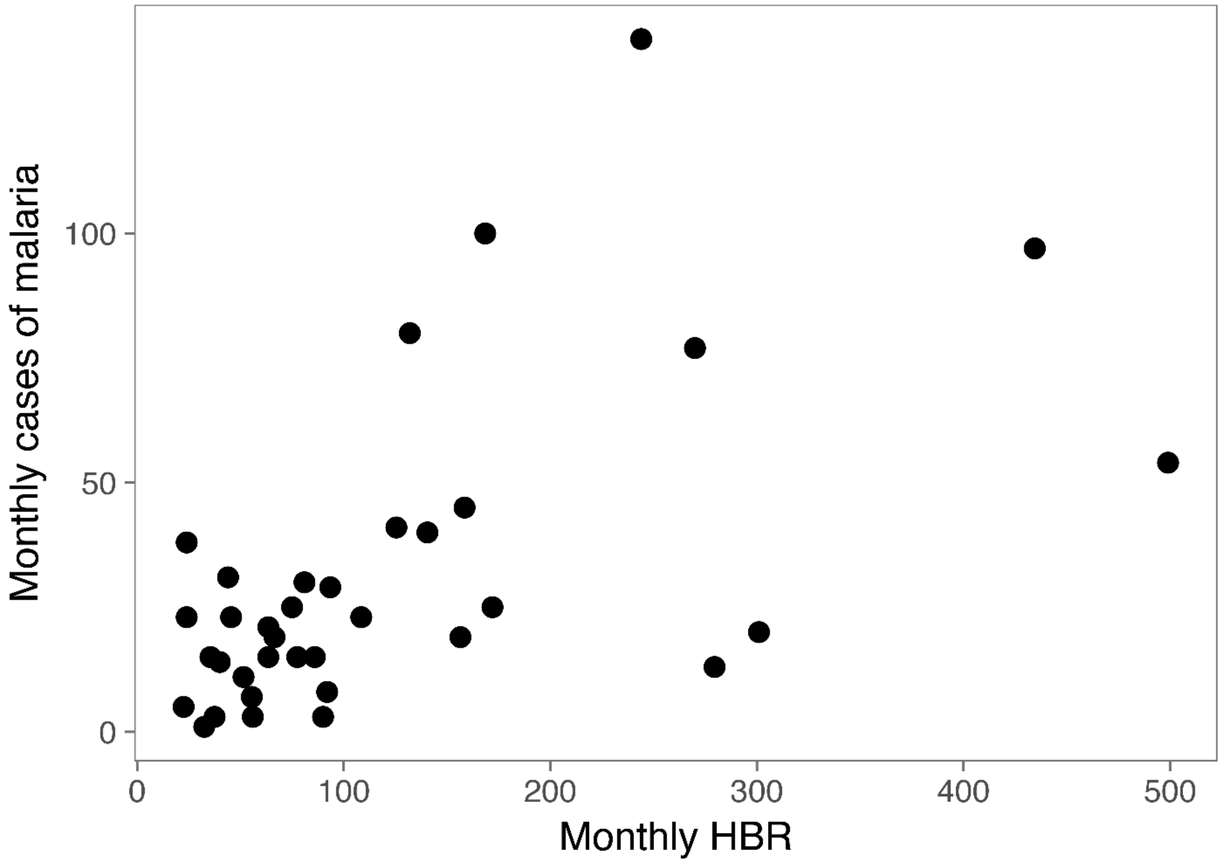

Figure S4. Estimation of the number of clusters in SNP dataset using STRUCTURE and Discriminant Analysis of Principal Components (DAPC). (A) Posterior probability of the data for STRUCTURE *K*=1 to 10. (B) STRUCTURE Δ*K* using Evanno method for *K*=2 to 9. (C) Bayesian Information Criterion (BIC) for 1 to 30 clusters using K-means clustering in preparation for DAPC.

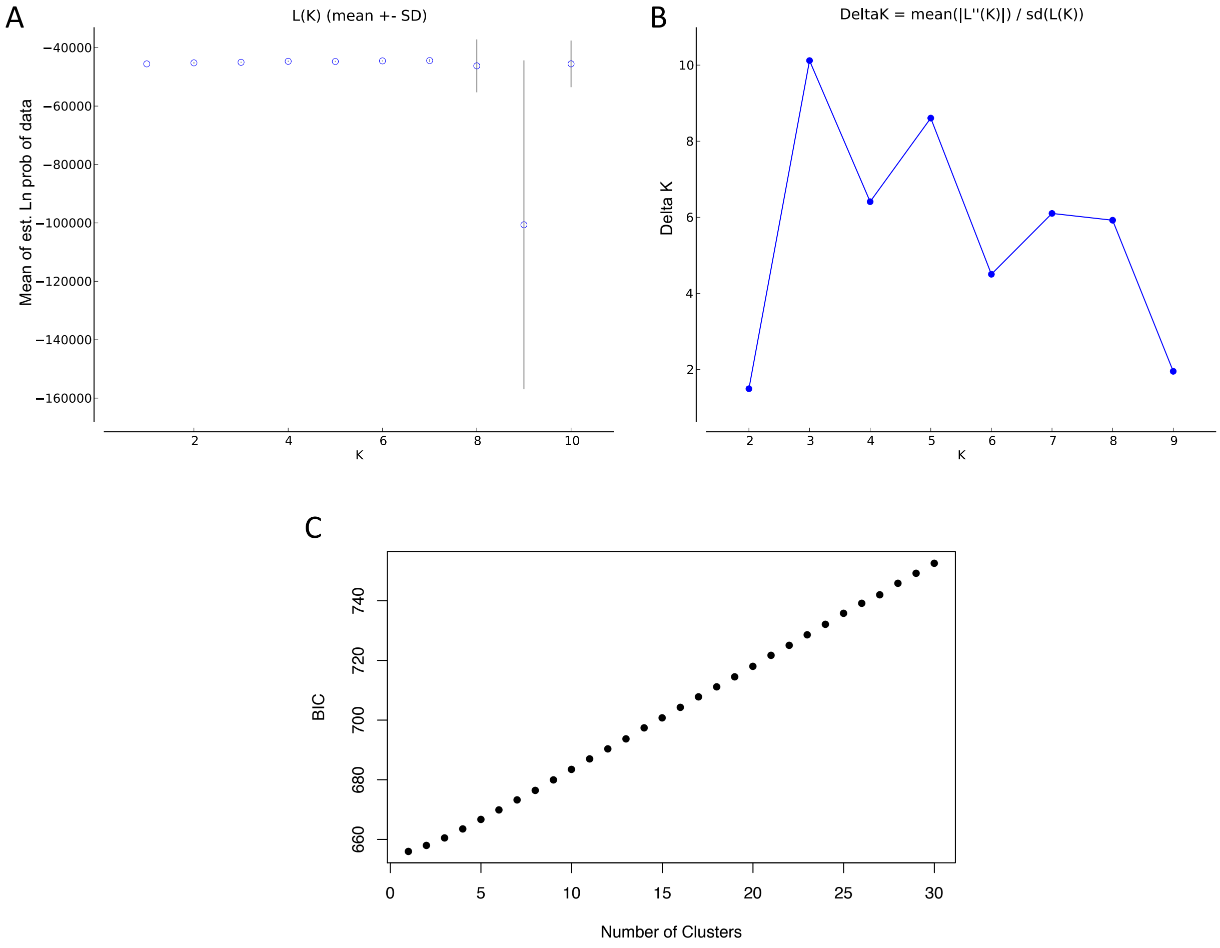

Figure S5. Results of STRUCTURE analysis of SNP dataset depicting two (A-C) and three (D-E) inferred genetic clusters, with individual *An. darlingi* ordered by locality/year (A & D), endophagic/exophagic (B), and collection time period (C & E).

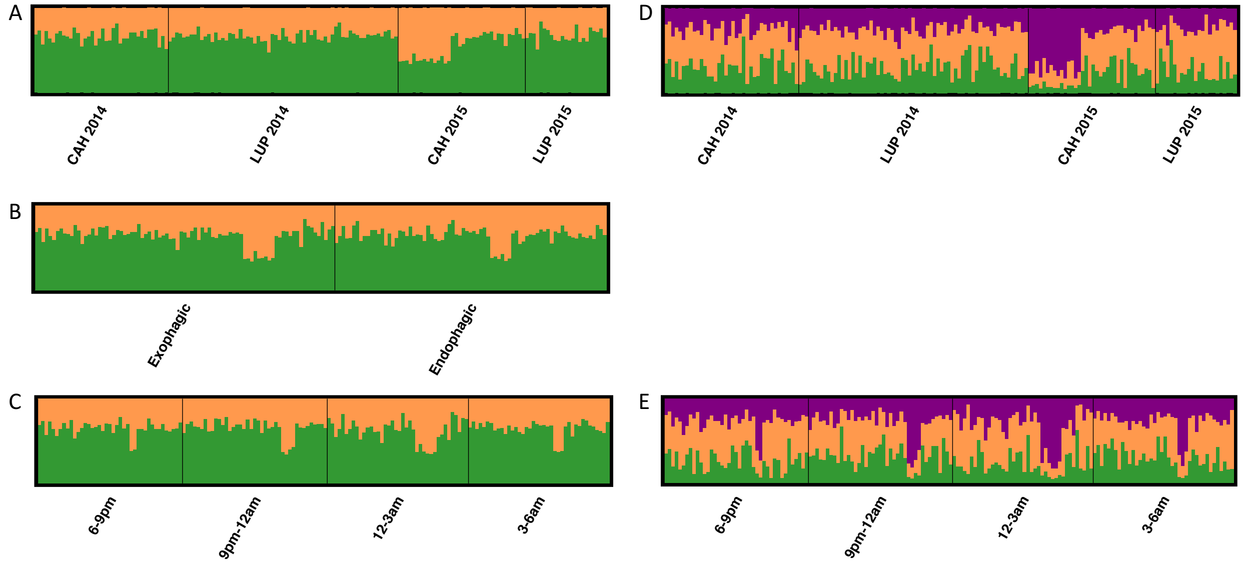

Figure S6. Results of Principal Components Analysis (PCA) of SNP dataset, with colors reflecting locality and year (A) or collection time period (B) of collected *An. darlingi*.

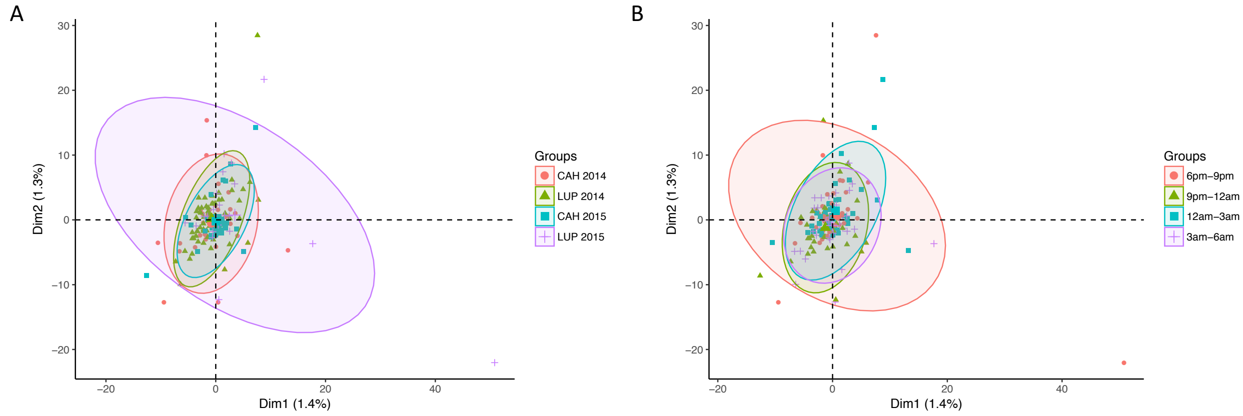

Supplement: Supplementary file 1 — Additional file 1: Table S1A. Monthly abundance, HBR, IR, EIR of Anopheles darlingi from Cahuide and Lupuna, 2013–2015. Table S1B. Monthly abundance, HBR, IR, EIR of Anopheles darlingi from Santa Emilia, 2015. Table S2. Monthly abundance, HBR, IR, EIR of Anopheles darlingi from Santa Emilia, 2014. Table S3. Infection of Anopheles darlingi by year, month, time, exophagic versus endophagic, village and Plasmodium species. Table S4. Kruskal-Wallis analysis on ranked abundance of Anopheles darlingi, Cahuide and Lupuna, 2013–2015 rainy season (January-June). Table S5. Pairwise FST values comparing Anopheles darlingi by (A) year/locality, (B) exophagic/endophagic, and (C) collection time. Figure S1. Schedule of Anopheles darlingi human landing catch collections in Lupuna, Cahuide, and Santa Emilia, 2013–2015. Figure S2. Number of reported human cases of Plasmodium vivax and Plasmodium falciparum in Cahuide, Lupuna, and Santa Emilia, 2010–2016. Figure S3. Monthly human biting rate plotted against the monthly number of malaria cases in Cahuide and Lupuna, rainy season, 2013–2015. Figure S4. Estimation of number of clusters in SNP dataset. Figure S5. Results of STRUCTURE analysis of SNP dataset for K=2 and K=3, with individual Anopheles darlingi ordered by locality/year, exophagic/endophagic, and collection time period. Figure S6. Results of PCA of SNP dataset, with individual Anopheles darlingi coloured by locality/year and collection time period. [file 12936_2018_2234_MOESM1_ESM.pdf]
